# Prevalence and Impact of Treatment-Resistant Depression in Latin America: a Prospective, Observational Study

**DOI:** 10.1007/s11126-021-09930-x

**Published:** 2021-08-31

**Authors:** Bernardo Soares, Gabriela Kanevsky, Chei Tung Teng, Rodrigo Pérez-Esparza, Gerardo Garcia Bonetto, Acioly L. T. Lacerda, Erasmo Saucedo Uribe, Rodrigo Cordoba, Christian Lupo, Aline Medeiros Samora, Patricia Cabrera

**Affiliations:** 1 Janssen Cilag-United Kingdom, Buckinghamshire, UK; 2 Janssen Cilag-Argentina, Buenos Aires, Argentina; 3 Department of Psychiatry, Institute of Psychiatry, University of São Paulo School of Medicine Clinics Hospital, São Paulo, Brazil; 4 Addiction Research Laboratory, Instituto Nacional de Neurologia y Neurocirugía, Mexico City, Mexico; 5 Investigaciones Clinicas-Instituto Medico DAMIC, Hospital Neuropsiquiatrico, Córdoba, Argentina; 6 PRODAF - Programa de Transtornos Afetivos and Laboratory of Integrative Neuroscience, Universidade Federal de São Paulo; and CNS Unit, BR Trials, São Paulo, Brazil; 7 Departamento de Psiquiatria, Centro de Neurociencias Avanzadas, Hospital Universitario, Universidad Autónoma de Nuevo León, Monterrey, Nuevo León, México; 8 Escuela de Medicina y Ciencias de la Salud, Universidad del Rosario; Centro Rosarista de Salud Mental; and Centro de Investigaciones del Sistema Nervioso - Grupo Cisne, Bogotá, Colombia; 9 Centro de Investigación y Asistencia en Psiquiatría, Rosario and National University of Rosario, Santa Fe, Argentina; 10 Janssen Cilag-Brazil, São Paulo, Brazil; 11 Janssen Cilag-Colombia, Bogotá, Colombia

**Keywords:** Treatment-resistant depressive disorder, Major depressive disorder, Prevalence, Health care utilization, Latin America

## Abstract

Approximately one-third of patients with major depressive disorder (MDD) have treatment-resistant depression (TRD). The TRAL study will evaluate the prevalence and impact of TRD among patients with MDD in four Latin American countries. In this multicenter, prospective, observational study, patients with MDD were recruited from 33 reference sites in Mexico, Colombia, Brazil, and Argentina. Patients were assessed for TRD, defined as failure to respond to ≥ 2 antidepressant medications of adequate dose and duration. Demographics, previous/current treatments, depressive symptoms, functioning, healthcare resource utilization, and work impairment were also collected and evaluated using descriptive statistics, chi-square test, Fisher exact test, *t*-test for independent samples, or the Mann–Whitney nonparametric test, as appropriate. 1475 patients with MDD were included in the analysis (mean age, 45.6 years; 78% women); 89% were receiving relevant psychiatric treatment. 429 patients met criteria for TRD, and a numerically higher proportion of patients with TRD was present in public versus private sites of care (31% vs 27%). The mean Montgomery-Asberg Depression Rating Scale score was 25.0 among all MDD patients and was significantly higher for patients with TRD versus non-TRD (29.4 vs 23.3; *P* < 0.0001). Patients with TRD, versus those with non-TRD, were significantly more likely to be older, have a longer disease duration, have more comorbidities, be symptomatic, have a higher median number of psychiatric consultations, and report greater work impairment. Patients with TRD have a disproportionate burden of disease compared to those with non-TRD. Appropriate treatment for TRD is a substantial unmet need in Latin America. https://www.ClinicalTrials.gov identifier NCT03207282, 07/02/2017.

**Supplementary Information:**

The online version contains supplementary material available at 10.1007/s11126-021-09930-x.

## Background

Major depressive disorder (MDD) is a worldwide health concern, affecting over 300 million people. MDD has a significant impact on core aspects of life, including sleeping, eating, intellect, and self-worth [1, 2]. In a recent systematic review and meta-analysis, 27.0% of outpatients had depression or showed depressive symptoms [3]. According to the World Health Organization, MDD is the largest contributor to disability globally [1]. The impact of MDD on disability is due, in part, to a substantial proportion of patients with MDD who do not respond to current treatments despite available antidepressants and augmentation strategies [4].

An estimated one-third of patients with MDD have treatment-resistant depression (TRD), usually defined as a failure to respond to ≥ 2 antidepressant medications of adequate dose and duration [5–7]. A great deal of MDD-related disease burden can be ascribed to TRD. Compared to patients with MDD who are not resistant to treatment, those with TRD have more comorbidities, poorer health-related quality of life, greater risk of suicide, greater direct and indirect healthcare resource utilization, and greater costs [8–14]. Patients with TRD currently have limited approved therapeutic options. For example, in the United States, only fluoxetine/olanzapine was approved for the treatment of TRD until March 2019, when intranasal esketamine was also approved [15–17].

Most studies of depression in Latin America have focused broadly on MDD. One study of MDD involving 1835 patients from Argentina, Brazil, Chile, Colombia, and Mexico hospital emergency departments showed an MDD prevalence ranging from 23.0% to 35.0% [18]. Compared to patients without MDD, those with MDD were more likely to be middle aged, be women, be smokers, have a lower socioeconomic level, and have a diagnosis of asthma or arthritis/rheumatism. Multivariate analysis identified an independent association between MDD and lower education level, smoking, self-reported anxiety, chronic fatigue, and back problems. Additionally, two Latin American, multicenter, observational studies found that the presence of somatic symptoms in patients with MDD was associated with higher depression severity and resulted in higher pain scores and a deleterious effect on quality of life [19]. The São Paulo Ageing & Health Study found that older Brazilian adults (≥ 65 years of age) with MDD had higher rates than nondepressed older adults of both hospitalization and use of outpatient services, underlining the healthcare burden of MDD in Latin America [20].

Although depression has been systematically studied in Latin America, TRD has not. The TRAL (Treatment Resistant Depression in *America Latina*) study is the first international, multicenter, prospective, observational, noninterventional study of TRD in four Latin American countries: Mexico, Colombia, Brazil, and Argentina. In this study, the diagnosis of MDD was determined by semi-structured interview to ensure a uniform study population, and patients found to have TRD will be followed for an additional year to gain insight into changes in responses over time. Reported here are results from the baseline cross-sectional analysis of TRAL.

## Materials and Methods

### Objectives

The primary objectives of this study are to 1) estimate the prevalence of TRD among patients with MDD being treated in psychiatric reference sites (clinic, ambulatory, hospital, day-hospital) in Mexico, Colombia, Brazil, and Argentina and 2) evaluate depression-related healthcare resource utilization among patients with TRD. As a secondary outcome, the study aims to describe the characteristics of patients with MDD, including comorbidities, treatment standards, severity of symptoms, utilization of medical resources, and level of disability.

### Study Design and Population

The TRAL study is an international, multicenter, prospective, observational, noninterventional study consisting of two phases:

1. Phase 1 (cross-sectional): patients with MDD were assessed to determine demographic data, previous and current treatments, depressive symptoms, suicidality, quality of life, functioning/disability, and general life. TRD prevalence was estimated, and patients with this diagnosis were included in Phase 2.
2. Phase 2 (cohort): 1-year follow up of a subset of patients with TRD.

Key inclusion criteria for Phase 1 were women and men; age ≥ 18 years; an MDD diagnosis according to the *Diagnostic and Statistical Manual of Mental Disorders, 5th Edition* and confirmed by the MINI International Neuropsychiatric Interview, 7.0.2 version (MINI); treatment or lack of treatment for a new or continued episode of depression at the time of enrollment; and the capability to complete the corresponding assessments in the study. The diagnosis of TRD was based on the following criteria: adequate follow-up and treatment with ≥ 2 antidepressants and lack of a complete response to treatment (based on the Montgomery-Asberg Depression Rating Scale [MADRS]); each investigator diagnosed TRD according to their discretion based on these criteria. Key exclusion criteria were a diagnosis of psychosis, schizophrenia, bipolar disorder, schizoaffective disorder, or dementia; substance dependence that was considered serious by the investigator; and current participation in another clinical study. Patients with a clinical diagnosis of depression, assessed by a single healthcare provider, were assessed for inclusion criteria in Phase 1. Data sources included patients’ medical records, as well as questionnaires, scales, and assessments completed by patients and investigators.

### Outcome Measures

The primary outcomes of the TRAL study were 1) the prevalence of TRD among MDD patients being treated in a psychiatric reference site and 2) depression-related healthcare resource utilization in TRD patients. Secondary outcomes included 1) TRD patients’ characteristics, including comorbid conditions, treatment patterns, severity of symptoms, and level of disability; 2) suicidality risk (ideation and attempts) in TRD patients; 3) total healthcare costs and depression-related healthcare costs in TRD patients; and 4) indirect costs associated with work productivity loss, daily functioning loss, quality of life, and caregiver burden. This interim analysis focuses on primary outcome 1 and secondary outcome 1.

### Analyses

Data collected included sociodemographic and clinical characteristics, clinical response (measured by MADRS total score), current disease status, MINI results, previous and current medication use, healthcare resource utilization, and work productivity (as measured by the Work Productivity and Activity Impairment Questionnaire: Depression [WPAI:D]). Prevalence of TRD was evaluated in the overall population and by country among patients with MDD (primary endpoint) and type of site of care (private or public). The proportion of untreated patients with MDD was also evaluated. Treated patients were defined as having received ≥ 1 current relevant psychiatric therapy by the first study visit. Other variables, including scales and questionnaires, were assessed among all patients with MDD, the subset of patients without TRD (non-TRD), and the subset of patients with TRD.

Descriptive statistics were used to evaluate sociodemographic and clinical variables in patients with MDD and by group (non-TRD and TRD) during Phase 1. For quantitative variables, mean, standard deviation (SD), median, and range were calculated. For qualitative variables, frequencies and percentages were calculated. 95% confidence intervals were also presented for prevalence.

Comparisons between TRD and non-TRD regarding categorical variables were performed using the chi-square test (CS) or Fisher exact test and through the *t*-test for independent samples (TT) or the Mann–Whitney nonparametric test (MW), according to the assumption validations of the statistical tests for quantitative variables, as identified in the tables. Comparison between countries regarding the proportion of treated patients was performed through the CS test. There was no imputation of missing data, except for incomplete dates. All statistical tests were two-tailed considering a significance level of 5%. Statistical analysis was conducted through the software SAS^®^ (version 9.4; SAS Institute Inc, Cary, NC, USA).

## Results

### Study Population and TRD Prevalence

A total of 1544 patients were screened, of whom 1475 (96%) were included in the analysis dataset for Phase 1. Patients were from 33 centers in four countries: Mexico (*n* = 697; 47% of all patients), Colombia (*n* = 162; 11%), Brazil (*n* = 396; 27%), and Argentina (*n* = 220; 15%). Among these patients with MDD, 89% were treated and 11% were untreated (Table 1). By country, the proportion of untreated patients ranged from 2% (Argentina) to 14% (Mexico), and differences between countries were statistically significant.

**Table 1:** Prevalence of treated and untreated MDD among patients, overall and by country^a^

	Total MDD population (*N* = 1475)	Mexico (*n* = 697)	Colombia (*n* = 162)	Brazil (*n* = 396)	Argentina (*n* = 220)	*P* value
Treated	1318 (89.4%)	601 (86.2%)	140 (86.4%)	362 (91.4%)	215 (97.7%)	
Untreated	157 (10.6%)	96 (13.8%)	22 (13.6%)	34 (8.6%)	5 (2.3%)	< 0.0001 (CS)

*MDD* major depressive disorder, *CS* chi-square test

^a^Patients were classified as treated if they had a “yes” answer for the question “Is the patient receiving psychiatric therapy?” at Visit 1

**Fig. 1:**
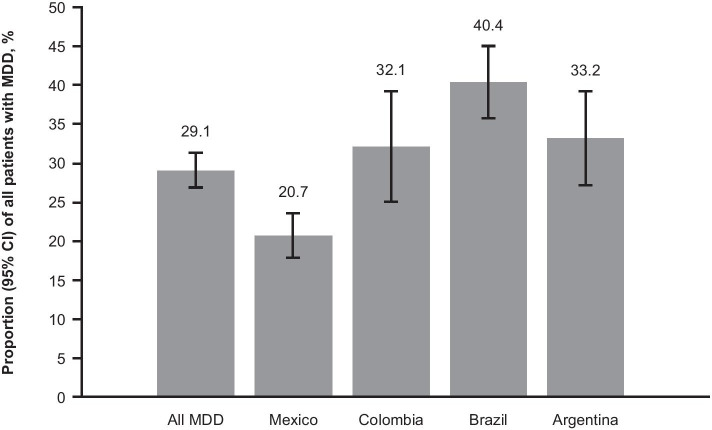
Prevalence of TRD among patients with MDD, overall and by country. *TRD* treatment-resistant depression, *MDD* major depressive disorder, *CI* confidence interval

The prevalence of TRD in Latin American sites among patients with MDD was 29% (429 patients; 95% confidence interval: 27%–31%; Fig. 1). Among treated patients with MDD, the prevalence of TRD was 32% (95% confidence interval: 29%–34%). By country, the lowest prevalence of TRD was observed in Mexico (21%) and the highest was observed in Brazil (40%); prevalences in Colombia and Argentina were 32% and 33%, respectively. Overall, the prevalence of TRD was numerically higher in public sites of care (31%) than in private sites of care (27%; Table 2), and varied by more specific categorizations (eg, 19% among patients in public psychiatric clinical sites vs 60% among patients at general hospitals; Online Resource 2).

**Table 2:** TRD prevalence among patients with MDD by type of care site, overall and by country^a^

	Total MDD population (*N* = 1475)	Mexico (*n* = 697)	Colombia (*n* = 162)	Brazil (*n* = 396)	Argentina (*n* = 220)
Private sites of care	202 (27.1%) [23.9%–30.3%]	78 (22.3%) [17.9%–26.6%]	41 (35.3%) [26.2%–44.0%]	10 (16.7%) [7.2%–26.1%]	73 (33.2%) [27.0%–39.4%]
Public sites of care	227 (31.1%) [27.8%–34.5%]	66 (19.0%) [14.9%–23.1%]	11 (23.9%) [11.6%–36.2%]	150 (44.6%) [39.3%–50.0%]	–

*TRD* treatment-resistant depression, *MDD* major depressive disorder, *CI* confidence interval

^a^Data are reported as prevalence (95% CI). Prevalence is based on the total numbers of patients from private or public sites of care, as appropriate. These values for private and public sites of care, respectively, were: 746 and 729 (total MDD population), 350 and 347 (Mexico), 116 and 46 (Colombia), 60 and 336 (Brazil), and 220 (Argentina [private sites of care only])

### Sociodemographic and Clinical Characteristics

Among all patients with MDD, the mean age was 45.6 years, 78% were female, 44% had ≥ 13 years of formal education, the mean age at diagnosis was 37.9 years, the median duration of MDD was 3.6 years, and 50% had a disease other than MDD (Table 3). Compared to patients with non-TRD, those with TRD were significantly older, were more likely to be divorced/separated, had a longer MDD disease duration, and were more likely to have comorbidities. No significant differences were observed between the non-TRD and TRD groups in regard to age at diagnosis, years of formal education, and number of hospitalizations for MDD in the past year.

**Table 3:** Sociodemographic and clinical characteristics at baseline

	All MDD (*N* = 1475)	Non-TRD *(n* = 1046)	TRD (*n* = 429)	*P* value^a^
Age, years				
Mean	45.6	44.4	48.5	< 0.0001 (MW)
Standard deviation	15.22	15.85	13.13	
Gender, *n* (%)				
Female	1150 (78.0%)	803 (76.8%)	347 (80.9%)	0.0832 (CS)
Male	325 (22.0%)	243 (23.2%)	82 (19.1%)	
Marital status, *n* (%)^b^	1459	1036	423	
Single	538 (36.9%)	392 (37.8%)	146 (34.5%)	0.0035 (CS)
Married/consensual union	665 (45.6%)	486 (46.9%)	179 (42.3%)	
Divorced/separated	176 (12.1%)	106 (10.2%)	70 (16.5%)	
Widower	80 (5.5%)	52 (5.0%)	28 (6.6%)	
Years of formal education, *n* (%)^b^	1357	965	392	
0	4 (0.3%)	3 (0.3%)	1 (0.3%)	0.8170 (CS)
1–4	51 (3.8%)	35 (3.6%)	16 (4.1%)	
5–9	309 (22.8%)	212 (22.0%)	97 (24.7%)	
10–12	391 (28.8%)	283 (29.3%)	108 (27.6%)	
≥ 13	602 (44.4%)	432 (44.8%)	170 (43.4%)	
Age at diagnosis, years^b^	1434	1020	414	
Mean	37.9	38.2	37.3	0.5973 (MW)
Standard deviation	15.06	15.46	14.02	
MDD disease duration, years^b^	1434	1020	414	
Median	3.6	1.9	8.0	< 0.0001 (MW)
Minimum	0.0	0.0	0.0	
Maximum	69.2	46.9	69.2	
Number of hospitalizations for MDD in the last year				
Median	1.0	1.0	1.0	0.5888 (MW)
Minimum	1.0	1.0	1.0	
Maximum	5.0	5.0	4.0	
Number of days hospitalized in the last year^b^	80	56	24	
Median	14.0	14.0	11.5	0.7685 (MW)
Minimum	1.0	1.0	1.0	
Maximum	157.0	157.0	96.0	
Comorbidities, *n* (%)				
Disease other than MDD	741 (50.2%)	468 (44.7%)	273 (63.6%)	< 0.0001 (CS)
Respiratory^c^	90 (12.2%)	55 (11.8%)	35 (12.8%)	0.6753 (CS)
Cardiovascular^c^	318 (43.0%)	179 (38.3%)	139 (50.9%)	0.0008 (CS)
Digestive^c^	168 (22.7%)	95 (20.3%)	73 (26.7%)	0.0450 (CS)
Endocrine	308 (41.6%)	177 (37.8%)	131 (48.0%)	0.0068 (CS)
Genitourinary^c^	108 (14.6%)	55 (11.8%)	53 (19.4%)	0.0045 (CS)
Hematopoietic^c^	41 (5.5%)	23 (4.9%)	18 (6.6%)	0.3385 (CS)
Musculoskeletal^c^	174 (23.5%)	98 (21.0%)	76 (27.8%)	0.0339 (CS)
Neurological^c^	179 (24.2%)	113 (24.2%)	66 (24.2%)	0.9948 (CS)
Sense organs^c^	63 (8.5%)	41 (8.8%)	22 (8.1%)	0.7346 (CS)
Skin and appendices^c^	57 (7.7%)	38 (8.1%)	19 (7.0%)	0.5622 (CS)

*MDD* major depressive disorder, *TRD* treatment-resistant depression, *MW* Mann–Whitney nonparametric test, *CS* chi-square test

^a^TRD versus non-TRD

^b^Some patients had missing data. Numbers of patients with available data in each population are specified in this row

^c^One patient (with non-TRD) was missing data

### Characterization of Patients’ MDD

**Fig. 2:**
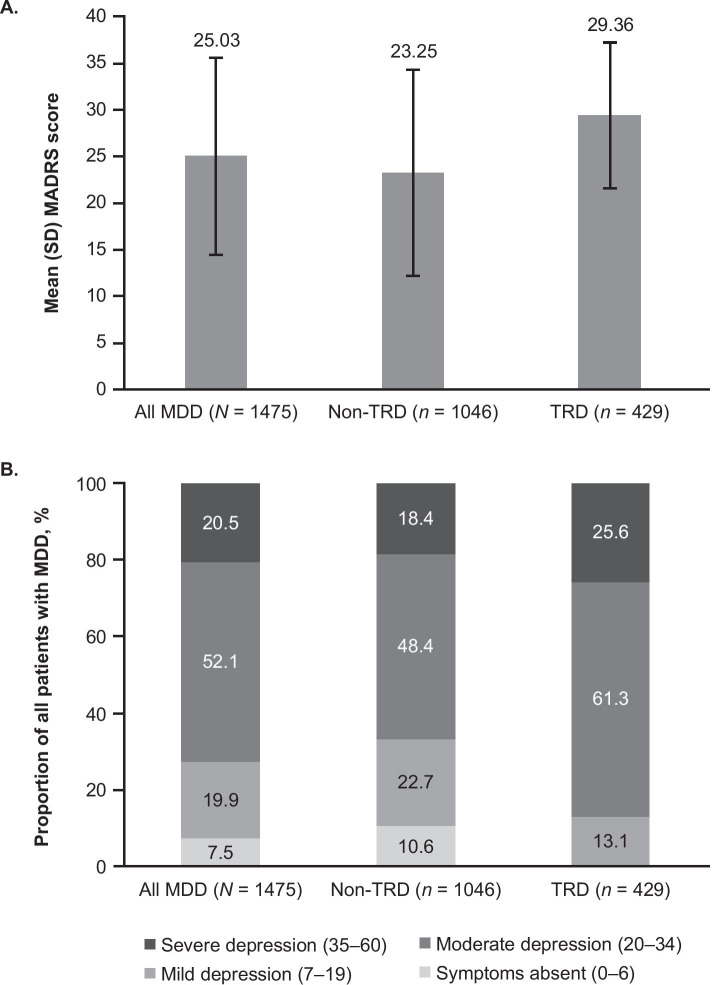
MADRS scores for patients with MDD, non-TRD, and TRD. **a** Mean (SD) total MADRS score. **b** Distribution of MADRS scores by severity group. *MADRS* Montgomery-Asberg Depression Rating Scale, *MDD* major depressive disorder, *TRD* treatment-resistant depression, *SD* standard deviation

The mean total MADRS score was 25.0 among all patients with MDD and was significantly lower for patients with non-TRD (23.3) versus those with TRD (29.4; *P* < 0.0001 [MW]; Fig. 2a). Significantly more patients with non-TRD (82%) had no symptoms or mild or moderate depression (MADRS total score 0–34) compared to patients with TRD (74%; *P* = 0.0016 [CS]). Among patients with TRD, 61% were classified as having moderate depression and 26% as having severe depression (Fig. 2b).

According to the current disease status questionnaire, a significantly higher proportion of patients with TRD (99.8%) were symptomatic compared to patients with non-TRD (90.3%; Online Resource 3). Significant differences between these groups were also observed for specific mental, emotional, or physical items (eg, persistent sad, anxious, or “empty” feelings; difficulty concentrating, remembering details, and making decisions). Notably, 39% of patients with TRD reported thoughts of suicide or suicide attempts (vs 25% of patients with non-TRD; *P* < 0.0001).

Based on MINI results, compared to patients with non-TRD, those with TRD were significantly more likely to have a current (91% vs 97%) or recurrent (43% vs 73%) major depressive episode, current suicidality (20% vs 35%) or a lifetime suicide attempt (14% vs 28%; all *P* < 0.0001), as well as generalized anxiety disorder (18% vs 26%; *P* = 0.0002; Table 4).

**Table 4:** Selected MINI results

	All MDD (*N* = 1475)	Non-TRD (*n* = 1046)	TRD (*n* = 429)	*P* value^a^
Major depressive episode				
Meets criteria, *n* (%)				
Current (2 weeks)	1366 (92.6%)	948 (90.6%)	418 (97.4%)	< 0.0001
Past	792 (53.7%)	533 (51.0%)	259 (60.4%)	0.0010
Recurrent	761 (51.6%)	449 (42.9%)	312 (72.7%)	< 0.0001
Suicidality				
Meets criteria, *n* (%)				
Current (past month)	358 (24.3%)	207 (19.8%)	151 (35.2%)	< 0.0001
Lifetime attempt	265 (18.0%)	143 (13.7%)	122 (28.4%)	< 0.0001
Low	195 (13.2%)	112 (10.7%)	83 (19.3%)	–
Moderate	67 (4.5%)	39 (3.7%)	28 (6.5%)	–
High	216 (14.6%)	125 (12.0%)	91 (21.2%)	–
Suicide behavior disorder				
Meets criteria, *n* (%)				
Current	96 (6.5%)	59 (5.6%)	37 (8.6%)	0.0349
In early remission	77 (5.2%)	37 (3.5%)	40 (9.3%)	< 0.0001
Panic disorder				
Meets criteria, *n* (%)				
Current (past month)	157 (10.6%)	109 (10.4%)	48 (11.2%)	0.6640
Lifetime	226 (15.3%)	128 (12.2%)	98 (22.8%)	< 0.0001
Agoraphobia				
Meets criteria, *n* (%)				
Current	136 (9.2%)	64 (6.1%)	72 (16.8%)	–
Social anxiety disorder (social phobia)				
Meets criteria, *n* (%)				
Current (past month)	131 (8.9%)	70 (6.7%)	61 (14.2%)	–
OCD				
Meets criteria, *n* (%)				
Current (past month)	68 (4.6%)	33 (3.2%)	35 (8.2%)	–
PTSD				
Meets criteria, *n* (%)				
Current (past month)	63 (4.3%)	35 (3.3%)	28 (6.5%)	0.0061
Alcohol use disorder				
Meets criteria, *n* (%)				
Past 12 months	40 (2.7%)	23 (2.2%)	17 (4.0%)	0.0582
Substance use disorder (nonalcohol)				
Meets criteria, *n* (%)				
Past 12 months	15 (1.0%)	6 (0.6%)	9 (2.1%)	0.0175^b^
Anorexia nervosa				
Meets criteria, *n* (%)				
Current (past 3 months)	5 (0.3%)	4 (0.4%)	1 (0.2%)	–
Bulimia nervosa				
Meets criteria, *n* (%)				
Current (past 3 months)	14 (0.9%)	7 (0.7%)	7 (1.6%)	–
Binge-eating disorder				
Meets criteria, *n* (%)				
Current (past 3 months)	26 (1.8%)	18 (1.7%)	8 (1.9%)	–
Generalized anxiety disorder				
Meets criteria, *n* (%)				
Current (past 6 months)	298 (20.2%)	185 (17.7%)	113 (26.3%)	0.0002
Antisocial personality disorder				
Meets criteria, *n* (%)				
Lifetime	18 (1.2%)	14 (1.3%)	4 (0.9%)	0.5189

*MINI* Mini International Neuropsychiatric Interview, *MDD* major depressive disorder, *TRD* treatment-resistant depression, *OCD* obsessive compulsive disorder, *PTSD* posttraumatic stress disorder

^a^Non-TRD versus TRD; *P* value calculated using a chi-square test, except as noted

^b^*P* value calculated using a Fisher exact test

### MDD Treatment Schemes

Among all patients with MDD, 63% had received previous psychiatric medication (non-TRD: 49%; TRD: 97%). Current therapy use among all patients with MDD was as follows: 89% of patients were on treatment with medications used for MDD (non-TRD: 86%; TRD: 97%) and 37% with other relevant therapy (ie, nonpsychiatric therapies prescribed for conditions other than MDD; non-TRD: 33%; TRD: 48%; Table 5). Eleven of the 429 patients with TRD were not currently being treated but were clinically considered to have TRD based on having MDD and a history of antidepressant failure. For these patients, the median (range) duration of time since the last psychiatric medication was 9.0 (0.0–208.0) months.

**Table 5:** Use of previous and current psychiatric therapies

	All MDD (*N* = 1475)	Non-TRD (*n* = 1046)	TRD (*n* = 429)
Previous psychiatric medication, *n* (%)	924 (62.6%)	508 (48.6%)	416 (97.0%)
Other previous relevant medication, *n* (%)	109 (7.4%)	64 (6.1%)	45 (10.5%)
Current relevant psychiatric therapy, *n* (%)	1318 (89.4%)	900 (86.0%)	418 (97.4%)
Current other relevant therapy, *n* (%)	551 (37.4%)	344 (32.9%)	207 (48.3%)
Duration of current treatments, months			
MAOIs, *n* (%)^a^	4 (0.3%)	1 (0.1%)	3 (0.7%)
Median	17.00	14.00	20.00
Minimum	0.00	14.00	0.00
Maximum	21.00	14.00	21.00
Missing^b^	0	0	0
Tricyclic antidepressants, *n* (%)^a^	29 (2.1%)	11 (1.2%)	18 (4.3%)
Median	7.50	4.00	10.00
Minimum	0.00	0.00	1.00
Maximum	122.00	122.00	75.00
Missing^b^	1	0	1
SSRIs, *n* (%)^a^	843 (62.4%)	617 (66.4%)	226 (53.6%)
Median	2.00	1.00	4.00
Minimum	0.00	0.00	0.00
Maximum	218.00	218.00	217.00
Missing^b^	28	17	11
SNRIs, *n* (%)^a^	335 (24.8%)	195 (21.0%)	140 (33.2%)
Median	2.00	2.00	3.00
Minimum	0.00	0.00	0.00
Maximum	203.00	203.00	140.00
Missing^b^	16	7	9
SDRIs, *n* (%)^a^	49 (3.6%)	19 (2.0%)	30 (7.1%)
Median	7.00	7.00	7.50
Minimum	0.00	0.00	0.00
Maximum	74.00	74.00	63.00
Missing^b^	4	0	4
Multimodal, *n* (%)^a^	39 (2.9%)	19 (2.0%)	20 (4.7%)
Median	3.00	1.00	3.00
Minimum	0.00	0.00	0.00
Maximum	25.00	25.00	12.00
Missing^b^	0	0	0
Antipsychotics, *n* (%)^a^	261 (19.3%)	128 (13.8%)	133 (31.5%)
Median	2.00	1.00	3.00
Minimum	0.00	0.00	0.00
Maximum	105.00	72.00	105.00
Missing^b^	21	7	14
Antiepileptics, *n* (%)^a^	133 (9.8%)	71 (7.6%)	62 (14.7%)
Median	10.00	11.00	9.50
Minimum	0.00	0.00	0.00
Maximum	355.00	302.00	355.00
Missing^b^	14	8	6
Brain stimulation techniques, *n* (%)^a^	2 (0.1%)	0 (0.0%)	2 (0.5%)
Median	3.00	–	3.00
Minimum	1.00	–	1.00
Maximum	5.00	–	5.00
Missing^b^	0	0	0
Psychotherapy, *n* (%)^a^	5 (0.4%)	1 (0.1%)	4 (0.9%)
Median	3.00	–	3.00
Minimum	1.00	–	1.00
Maximum	5.00	–	5.00
Missing^b^	2	0	2
Others, *n* (%)^a^	12 (0.9%)	4 (0.4%)	8 (1.9%)
Median	0.50	0.50	0.50
Minimum	0.00	0.00	0.00
Maximum	47.00	1.00	47.00
Missing^b^	0	0	0
Current use of ketamine/esketamine, *n* (%)^a^	1 (0.1%)	0 (0.0%)	1 (0.2%)
Previous use of ketamine/esketamine, *n* (%)^c^	9 (1.0%)	0 (0.0%)	9 (2.2%)
Current use of lithium, *n* (%)^a^	50 (3.7%)	11 (1.2%)	39 (9.2%)
Previous use of lithium, *n* (%)^c^	47 (5.0%)	10 (1.9%)	37 (8.9%)

*MDD* major depressive disorder, *TRD* treatment-resistant depression, *MAOI* monoamine oxidase inhibitor, *SSRI* selective serotonin reuptake inhibitor, *SNRI* serotonin and noradrenaline reuptake inhibitor, *SDRI* serotonin and dopamine reuptake inhibitor

^a^Percentages were calculated using the total number of patients using current relevant psychiatric therapy or current other relevant therapy (1351 patients among all patients with MDD)

^b^Number of patients who were missing data for the calculation of treatment duration

^c^Percentages were calculated using the total number of patients with data for previous use of ketamine/esketamine and/or lithium (935 patients among all patients with MDD)

The class of antidepressants most frequently reported as current medication was selective serotonin reuptake inhibitors (SSRIs; 62% of all patients), followed by serotonin and noradrenaline reuptake inhibitors (25%) and antipsychotics (19%). The proportion of patients currently using each class of therapy was numerically higher for the TRD group compared to the non-TRD group, with the exception of SSRIs. The median duration of current treatments was numerically longer for each class of therapy among patients in the TRD versus non-TRD group, with the exception of antiepileptics.

### Healthcare Resource Utilization and Work Productivity

Overall, 59% of patients with MDD had received ambulatory care (non-TRD: 57%; TRD: 66%); among these patients, 43% received up to seven days of care (non-TRD: 54%; TRD: 19%; Table 6). The median number of psychiatric consultations was significantly higher for patients with TRD (5) compared to patients with non-TRD (2). Based on the WPAI:D, in the previous seven days, depression led to a median of 13% of work time missed, 50% impairment while working, 58% overall work impairment, and 60% activity impairment. Significantly greater impairment was reported for patients with TRD versus non-TRD for the latter three items.

**Table 6:** Healthcare resource utilization and work productivity

	All MDD (*N* = 1475)	Non-TRD *(n* = 1046)	TRD (*n* = 429)	*P* value^a^
Ambulatory care, *n* (%)	877 (59.5%)	594 (56.8%)	283 (66.0%)	
Number of days, *n* (%)^b^				
≤ 7	375 (42.9%)	321 (54.1%)	54 (19.1%)	
> 7 to ≤ 30	280 (32.0%)	166 (28.0%)	114 (40.4%)	
> 30 to ≤ 60	58 (6.6%)	31 (5.2%)	27 (9.6%)	
> 60 to ≤ 90	143 (16.3%)	66 (11.1%)	77 (27.3%)	
> 90	19 (2.2%)	9 (1.5%)	10 (3.5%)	
Number of ED visits, *n*	826	563	263	
Median	0.00	0.00	0.00	
Minimum	0.00	0.00	0.00	
Maximum	30.00	30.00	20.00	
Number of psychiatrist consultations, *n*	863	587	276	< 0.0001
Median	2.00	2.00	5.00	
Minimum	0.00	0.00	0.00	
Maximum	64.00	60.00	64.00	
Number of psychologist consultations, *n*	819	561	258	
Median	0.00	0.00	0.00	
Minimum	0.00	0.00	0.00	
Maximum	55.00	50.00	55.00	
Number of other specialist consultations, *n*	807	551	256	
Median	0.00	0.00	0.00	
Minimum	0.00	0.00	0.00	
Maximum	72.00	30.00	72.00	
Number of primary care physician consultations, *n*	806	553	253	
Median	0.00	0.00	0.00	
Minimum	0.00	0.00	0.00	
Maximum	24.00	24.00	24.00	
Number of other health professional consultations, *n*	803	551	252	
Median	0.00	0.00	0.00	
Minimum	0.00	0.00	0.00	
Maximum	44.00	28.00	44.00	
Number of nonpharmaceutical treatment consultations, *n*	800	550	250	
Median	0.00	0.00	0.00	
Minimum	0.00	0.00	0.00	
Maximum	14.00	3.00	14.00	
WPAI:D				
Percent work time missed due to depression, *n*	596	459	137	0.3813
Median	12.77	11.11	17.65	
Minimum	0.00	0.00	0.00	
Maximum	100.00	100.00	100.00	
Percent impairment while working due to depression, *n*	529	407	122	0.0036
Median	50.00	50.00	60.00	
Minimum	0.00	0.00	0.00	
Maximum	100.00	100.00	100.00	
Percent overall work impairment due to depression, *n*	528	407	121	
Median	58.17	55.00	64.00	0.0025
Minimum	0.00	0.00	0.00	
Maximum	100.00	100.00	100.00	
Percent activity impairment due to depression, *n*	1473	1045	428	
Median	60.00	50.00	70.00	< 0.0001
Minimum	0.00	0.00	0.00	
Maximum	100.00	100.00	100.00	

*MDD* major depressive disorder, *TRD* treatment-resistant depression, *ED* emergency department, *WPAI:D* Work Productivity and Activity Impairment Questionnaire: Depression

^a^Non-TRD versus TRD; *P* value calculated using a Mann–Whitney nonparametric test

^b^Two patients had missing data (one non-TRD and one TRD)

## Discussion

Across four Latin American countries, 29% of patients with MDD were resistant to treatment, with TRD prevalences of 21% in Mexico, 32% in Colombia, 33% in Argentina, and 40% in Brazil. In comparison, the STAR*D trial, which enrolled patients with MDD who were candidates for medication as a first treatment step, found that approximately one-third of patients with MDD in the United States were treatment-resistant [5]. Other estimates of TRD in the United States have been lower (7%–12%), though, unlike TRAL, diagnosis of MDD and TRD was determined using a retrospective claims database [21, 22]. In Europe, a large multicenter study (European Group for the Study of Resistant Depression) found a TRD prevalence rate of 41% among patients with MDD, while a UK study of patients being treated for MDD in a primary care setting found that as many as 55% had TRD [12, 23]. Prevalence rates of TRD in other geographic regions have been estimated at 22% of patients in Canada receiving antidepressant treatment for MDD from a primary care physician; 21% of patients in Taiwan with new-onset, pharmaceutically treated MDD; and 12% of patients in Japan with new-onset, pharmaceutically treated MDD during a 1-year period of time [24–26]. Importantly, definitions of TRD varied across these studies, limiting direct comparison. The variation in TRD prevalence by country is further discussed below, but it is notable that there may be greater reluctance to report and seek treatment for depression and, by extension, TRD, among patients in East Asian countries.

In this interim analysis of TRAL, while the prevalence of TRD was similar in private and public sites of care overall, numerical differences were observed in some countries. In Colombia, 35% of patients in private settings had TRD versus 24% of patients in public settings; in contrast, in Brazil, 17% of patients in private settings had TRD versus 45% of patients in public settings. The higher prevalence of TRD in Brazilian public settings could be due to the nature of these public services, most of which are university-based research centers, with a greater demand from higher-complexity and more severe patients; however, further information is needed to confirm this assumption. When sites of care were examined in more detail among all patients with MDD, the site with the highest prevalence of TRD was the general hospital setting (60%). Notably, variability in access to healthcare may limit comparisons across countries. Variability in the types of care settings that participated in the current study may also limit interpretation of these results.

This interim analysis identified several concerning demographic characteristics of patients in Latin American with MDD. The mean age at which MDD was diagnosed was 37.9 years overall, 38.2 years for patients with non-TRD, and 37.3 years for patients with TRD, suggesting that earlier diagnosis of MDD in Latin American countries is important. Earlier diagnosis could lead to earlier treatment, better outcomes for patients, and potentially a decreased burden of disease for patients and caregivers [27]. Additionally, more women than men were diagnosed with MDD. This is consistent with global reports of depression prevalence that have demonstrated that female sex is a significant risk factor for depression [28].

Previous studies have found higher hospitalization rates and lengths of stay for patients with TRD compared to those with non-TRD [9, 13]; however, no such associations were observed in the interim analysis of the current study. This finding may reflect economic and cultural differences between Latin America and higher-income countries. In Latin America, patients with MDD may face external challenges accessing mental healthcare, as well as stigma associated with seeking care for mental health. Moreover, it is important to note that the current analysis includes only data from the baseline study visit, and hospitalization information was taken retrospectively. Healthcare resource utilization will also be evaluated in the 1-year longitudinal phase of TRAL; follow-up during this phase will include direct collection of hospitalization information and thus may provide more accurate information than that collected in Phase 1.

A higher mean MADRS total score was observed in the TRD group (29.4; SD: 7.9) than in the non-TRD group (23.3; SD: 11.2). Among patients with TRD, 87% had moderate or severe depression; however, the relatively high proportion of patients with TRD who were classified as having moderate depression (61%) compared to severe depression (26%) was surprising. This indicates that the greatest proportion of unmet need for patients in Latin America with TRD may be in treatment of moderate depression.

Based on current disease status items and the MINI, numerous factors were significantly more common among patients with TRD versus non-TRD, including suicidality and anxiety. This is in agreement with other published data; a large European multicenter study showed an association between suicidality and treatment resistance [12], and other studies have demonstrated associations between TRD and comorbid anxiety disorders [29, 30]. A systematic review of socio-demographic and clinical predictors of TRD found that a current or lifetime diagnosis of generalized anxiety disorder was predictive of nonresponse to depression treatment, while anxious symptoms, irrespective of a diagnosis, influenced remission from depression [28]. Further, the presence of more than 1 anxiety disorder in a single patient is also associated with TRD [29].

Compared to patients with non-TRD, numerically higher proportions of those with TRD had taken a previous psychiatric medication or were currently receiving relevant psychiatric therapy. Use of numerous classes of treatment were observed among patients with TRD, although therapies such as brain stimulation techniques (1%) and ketamine/esketamine (< 1% current use; 2% previous use) were low, potentially due to difficulty accessing them. Notably, for some treatment classes, many patients had missing data (the exact number varied by treatment class). This is likely due to patients not remembering previous treatments or the correct dates or doses of previous treatment regimens.

While TRD has been associated with increased healthcare resource utilization [9, 31], the only significant difference observed in the current study was a higher number of psychiatrist consultations for patients with TRD in comparison to non-TRD. As discussed previously, this may be due, at least in part, to difficulty of access and cultural sensitivities around seeking help for mental health issues in Latin America. As expected based on previous studies [9, 10], patients with TRD demonstrated significantly greater work impairment than patients with non-TRD on most WPAI:D items.

One of the strengths of this study is the quality of the diagnosis of MDD, which was defined, in part, using the semi-structured interview, MINI. Many TRD studies have defined MDD using presumptive diagnoses from patient registry databases of public or private health services. This more direct MDD diagnosis ensures a more uniform study population and thus the potential to detect more subtle differences between groups. Importantly, the present analysis represents baseline results; further information will be reported upon study completion.

The present study is not a population-based survey, as it included only individuals being assisted in clinical services (clinics, hospitals, community services) that treat mental disorders, independent of whether they are specialized or not. This could be perceived as a limitation for a prevalence study, considering that many cases of depression go undiagnosed in a general medicine setting. However, it was the authors’ decision to investigate the prevalence of treatment resistance among those diagnosed with MDD and to investigate predictors of TRD and differences between TRD and non-TRD populations.

## Conclusion

Present findings demonstrate that TRD represents a disproportional economic and social burden to healthcare systems, patients, and their families, and continues to be a substantial unmet need in the treatment of depression, including in Latin America.

## Supplementary Information

Below is the link to the electronic supplementary material.

Supplementary file1 (PDF 309 KB)

Supplementary file2 (PDF 292 KB)

Supplementary file3 (PDF 314 KB)

## Data Availability

The data sharing policy of Janssen Pharmaceutical Companies of Johnson & Johnson is available at https://www.janssen.com/clinical-trials/transparency. As noted on this site, requests for access to the study data can be submitted through the Yale Open Data Access (YODA) Project site at http://yoda.yale.edu.
