# Supplementary material for: Prevalence and Impact of Treatment-Resistant Depression in Latin America: a Prospective, Observational Study
Source: Psychiatr Q. 2021 Aug 31;92(4):1797–815. doi: 10.1007/s11126-021-09930-x (PMC8531108; doi:10.1007/s11126-021-09930-x)
Supplement: Supplementary file 1 — Supplementary file1 (PDF 309 KB) [file 11126_2021_9930_MOESM1_ESM.pdf]

Prevalence and impact of treatment-resistant depression in Latin America: a prospective, observational study

Psychiatric Quarterly

Bernardo Soares, Gabriela Kanevsky, Chei Tung Teng, Rodrigo Pérez-Esparza, Gerardo Garcia Bonetto, Acioly L.T. Lacerda, Erasmo Saucedo Uribe, Rodrigo Cordoba, Christian Lupo, Aline Medeiros Samora, Patricia Cabrera

Correspondence: Patricia Cabrera
Current affiliation: Janssen Global Services, Inc, Titusville, NJ
1125 Trenton-Harbourton Road, Titusville, NJ 08560
Telephone: +1 (609) 730-3180
Email: pcabrer1@ITS.JNJ.com

Online Resource 1 Listing of local ethics committees and institutional review boards

**Country**	**Name**
Brazil	CEP HUPES
	Comitê de Ética em Pesquisa do Hospital Universitário Walter Cantídio
	COEP-UFMG
	CEP- INC - Instituto de neurologia de Curitiba
	Comite de Etica em pesquisa IPUB-UFRJ
	Comitê de Ética em Pesquisa Investiga – Institutos de Pesquisa
	Comitê de Ética em Pesquisa em Seres Humanos da Faculdade de Medicina da Universidade Federal de Pelotas
	Comissão de Ética para Análise de Projetos de Pesquisa - CAPPesq
	Comitê de Ética em Pesquisa Hospital São José
	Comitê de Ética em Pesquisado Hospital de Clínicas de Porto Alegre (CEP / HCPA)
Colombia	C.E.I. Campo Abierto LTDA
	E.S.E. Hospital Mental de Antioquia
	Clínica CEIC de la Fundación Centro de Investigación Clínica, CIC
Argentina	Comité Independiente de Ética de Investigación en Salud Prof. Dr. Marcelino Rusculleda

	Comité de Bioética e Investigación de la Fundación para el Estudio y Tratamiento de las Enfermedades Mentales (FETEM)
	CAICI – CIAP<br><br>Instituto Centralizado de Asistencia e Investigación Clínica Integral – Centro de Investigación y Asistencia en Psiquiatría
	Instituto Médico Platense S.A.
	Comité Independiente de Ética para Ensayo en Farmacología Clínica. Fundación de Estudios Farmacológicos y de Medicamentos Prof. Luis M. Zeiher
Mexico	1. Sanatorio Alcocer Pozo S.A. de C.V.<br>2. Investigación Biomédica para el Desarrollo de Fármacos, S.A. de C.V.
	Instituto Nacional de Neurología y Neurocirugía Manuel Velazco Suárez
	Hospital La Misión, S.A. de C.V.
	Instituto Nacional de Psiquiatría Ramón de la Fuente Muñiz
	Hospital Central Dr. Ignacio Morones Prieto
	Comité Institucional de Ética en Investigación- ISSSTE
