# Supplementary material for: Prevalence and Impact of Treatment-Resistant Depression in Latin America: a Prospective, Observational Study
Source: Psychiatr Q. 2021 Aug 31;92(4):1797–815. doi: 10.1007/s11126-021-09930-x (PMC8531108; doi:10.1007/s11126-021-09930-x)
Supplement: Supplementary file 2 — Supplementary file2 (PDF 292 KB) [file 11126_2021_9930_MOESM2_ESM.pdf]

Prevalence and impact of treatment-resistant depression in Latin America: a prospective, observational study

Psychiatric Quarterly

Bernardo Soares, Gabriela Kanevsky, Chei Tung Teng, Rodrigo Pérez-Esparza, Gerardo Garcia Bonetto, Acioly L.T. Lacerda, Erasmo Saucedo Uribe, Rodrigo Cordoba, Christian Lupo, Aline Medeiros Samora, Patricia Cabrera

Correspondence: Patricia Cabrera
Current affiliation: Janssen Global Services, Inc, Titusville, NJ
1125 Trenton-Harbourton Road, Titusville, NJ 08560
Telephone: +1 (609) 730-3180
Email: pcabrer1@ITS.JNJ.com

Online Resource 2 Prevalence (95% CI) of TRD among patients with MDD by type of site of care

General hospital	95/158 (60.1%) [52.5%–67.8%]
General psychiatric hospital	14/40 (35.0%) [20.2%–49.8%]
Private clinical site	90/412 (21.8%) [17.9%–25.8%]
Private psychiatric clinical site	82/270 (30.4%) [24.9%–35.9%]
Private psychiatric institution	26/46 (56.5%) [42.2%–70.8%]
Public hospital	48/212 (22.6%) [17.0%–28.3%]
Public psychiatric clinical site	13/68 (19.1%) [9.8%–28.5%]
Public psychiatric hospital	61/269 (22.7%) [17.7%–27.7%]

CI, confidence interval; TRD, treatment-resistant depression; MDD, major depressive disorder.
