# Supplementary material for: Prevalence and Impact of Treatment-Resistant Depression in Latin America: a Prospective, Observational Study
Source: Psychiatr Q. 2021 Aug 31;92(4):1797–815. doi: 10.1007/s11126-021-09930-x (PMC8531108; doi:10.1007/s11126-021-09930-x)
Supplement: Supplementary file 3 — Supplementary file3 (PDF 314 KB) [file 11126_2021_9930_MOESM3_ESM.pdf]

**Prevalence and impact of treatment-resistant depression in Latin America: a prospective, observational study**

***Psychiatric Quarterly***

Bernardo Soares, Gabriela Kanevsky, Chei Tung Teng, Rodrigo Pérez-Esparza, Gerardo Garcia Bonetto, Acioly L.T. Lacerda, Erasmo Saucedo Uribe, Rodrigo Cordoba, Christian Lupo, Aline Medeiros Samora, Patricia Cabrera

Correspondence: Patricia Cabrera
Current affiliation: Janssen Global Services, Inc, Titusville, NJ
1125 Trenton-Harbourton Road, Titusville, NJ 08560
Telephone: +1 (609) 730-3180
Email: pcabrer1@ITS.JNJ.com

**Online Resource 3** Significant differences in current disease status among patients with non-TRD versus patients with TRD[a]

| Current status, n (%) | All MDD (N = 1475) | Non-TRD (n = 1046) | TRD (n = 429) | *P* value[b] |
|---|---|---|---|---|
| Persistent sad, anxious, or "empty" feelings | 1313 (89.0%) | 890 (85.1%) | 423 (98.6%) | <0.0001 |
| Feelings of hopelessness and/or pessimism | 1192 (80.8%) | 803 (76.8%) | 389 (90.7%) | <0.0001 |
| Feelings of guilt, worthlessness, and/or helplessness | 1149 (77.9%) | 793 (75.8%) | 356 (83.0%) | 0.0026 |
| Irritability, restlessness[c] | 1105 (75.2%) | 757 (72.6%) | 348 (81.5%) | 0.0004 |
| Loss of interest in activities or hobbies once pleasurable, including sex | 1273 (86.3%) | 863 (82.5%) | 410 (95.6%) | <0.0001 |
| Fatigue and decreased energy | 1316 (89.2%) | 901 (86.1%) | 415 (96.7%) | <0.0001 |

| Current status, n (%) | All MDD (N = 1475) | Non-TRD (n = 1046) | TRD (n = 429) | *P* value[b] |
|---|---|---|---|---|
| Difficulty concentrating, remembering details, and making decisions | 1214 (82.3%) | 819 (78.3%) | 395 (92.1%) | <0.0001 |
| Insomnia, early-morning wakefulness, or excessive sleeping[d] | 1123 (76.2%) | 767 (73.4%) | 356 (83.0%) | 0.0001 |
| Overeating or appetite loss | 1000 (67.8%) | 677 (64.7%) | 323 (75.3%) | 0.0001 |
| Thoughts of suicide, suicide attempts | 426 (28.9%) | 260 (24.9%) | 166 (38.7%) | <0.0001 |
| Persistent aches or pains, headaches, cramps, or digestive problems that do not ease even with treatment | 707 (47.9%) | 463 (44.3%) | 244 (56.9%) | <0.0001 |
| Current status | | | | |
| Asymptomatic | 102 (6.9%) | 101 (9.7%) | 1 (0.2%) | <0.0001 |
| Symptomatic | 1373 (93.1%) | 945 (90.3%) | 428 (99.8%) | |

TRD, treatment-resistant depression; MDD, major depressive disorder.

[a]Only current status items for which a statistically significant difference was observed between the non-TRD versus TRD groups are shown.

[b]Non-TRD versus TRD; *P* value calculated using a chi-square test.

[c]Six patients were missing data (four with non-TRD and two with TRD).

[d]One patient was missing data (non-TRD).
